# Peripheral blood transcriptomic signature is associated with cognitive impairment and freezing of gait in Parkinson’s disease: a data-driven approach

**DOI:** 10.1038/s41531-026-01527-0

**Published:** 2026-08-15

**Authors:** Reeree Lee, Mincheol Park

**Affiliations:** 1 Department of Nuclear Medicine, Chung-Ang University College of Medicine, Chung-Ang University Gwangmyeong Hospital, Gwangmyeong, Republic of Korea; 2 Division of Nuclear Medicine and Molecular Imaging, Department of Radiology, Stanford University, Stanford, USA; 3 Department of Neurology, Chung-Ang University College of Medicine, Chung-Ang University Gwangmyeong Hospital, Gwangmyeong, Republic of Korea

**Keywords:** Biomarkers, Diseases, Neurology, Neuroscience

## Abstract

Parkinson’s disease (PD) is a heterogeneous neurodegenerative disorder with variable long-term outcomes. Blood-based biomarkers for prognostic prediction remain underdeveloped. We aimed to develop a peripheral blood transcriptomic signature for predicting long-term complications in PD using unsupervised data-driven methods. Using RNA sequencing data from 541 PD patients and 180 healthy controls from the Parkinson’s Progression Markers Initiative (PPMI), we employed weighted gene correlation network analysis (WGCNA) to identify disease-associated gene modules. Contrastive principal component analysis was applied to derive a pseudo-temporal (PT) trajectory score reflecting molecular disease progression. The prognostic value of PT scores was assessed through Cox regression for cognitive impairment, freezing of gait (FOG), wearing-off, and levodopa-induced dyskinesia. WGCNA identified two PD-associated co-expression modules enriched for immune/inflammatory pathways. PT scores derived from these modules showed significant correlations with cognitive, autonomic, and axial motor symptoms. In multivariable Cox regression, higher PT scores independently predicted cognitive impairment (HR = 7.31, *P* = 0.002) and FOG (HR = 3.43, *P* < 0.001), but not predominantly dopaminergic motor complications. These findings demonstrate that peripheral blood transcriptomic signatures capture aspects of PD pathophysiology that are not fully explained by nigrostriatal dopaminergic degeneration alone, serving as potential prognostic biomarkers for cognitive impairment and FOG.

## Introduction

Parkinson’s disease (PD) is the second most common neurodegenerative disorder, characterized by distinctive motor symptoms such as bradykinesia, rigidity, and tremor, as well as various non-motor symptoms^1^. PD is a complex and heterogeneous disorder with several clinical phenotypes, ranging from tremor-predominant to akinetic-rigid motor subtypes^2^, while overall presentations vary from mild motor predominant to diffuse malignant subtypes^3^. Additionally, PD is often classified into subtypes based on associated neurotransmitter involvement, including noradrenergic subtypes^4^. Similarly, the pathomechanisms that cause and promote the disease are diverse, involving molecular and cellular processes such as perturbed alpha-synuclein proteostasis, impaired autophagy, mitochondrial dysfunction, oxidative stress, and neuroinflammation^5^. This complexity has hindered the development of widely accepted biomarkers for diagnosing, subtyping, and predicting prognosis in PD.

Long-term complications of PD significantly impact patient quality of life and represent important clinical endpoints for prognostic assessment. Motor fluctuations, including wearing-off (WO) phenomena and levodopa-induced dyskinesia (LID), are closely linked to dopaminergic medication use and nigrostriatal pathology, affecting up to 50% of patients within five years of levodopa initiation^6^. Freezing of gait (FOG) and cognitive impairment, however, are thought to involve additional pathomechanisms beyond dopaminergic dysfunction, including cholinergic and noradrenergic deficits, and are associated with a worse overall prognosis^7^. These distinct pathophysiological substrates indicate that different biomarkers may be required to predict predominantly dopaminergic versus predominantly non-dopaminergic complications.

However, biomarkers capable of predicting these long-term complications in PD remain limited. An ideal biomarker would facilitate precise differential diagnosis, exhibit high diagnostic sensitivity, accurately predict prognosis, and monitor disease progression^8^. While dopamine transporter (DaT) imaging may reflect nigrostriatal dopaminergic neuronal loss^9,10^, there exists controversy regarding the association between DaT imaging and nigrostriatal neuronal population^11,12^. Furthermore, DaT imaging does not directly reflect the underlying pathophysiology or burden of alpha-synucleinopathy^10^. Moreover, its high cost and limited accessibility pose challenges for widespread clinical use^13^. Recent research has focused on biofluid biomarkers. Cerebrospinal fluid (CSF) alpha-synuclein seed amplification assays (SAA) have demonstrated promising diagnostic value and the capacity to reflect the burden of alpha-synuclein-associated neuropathology^14^. However, the invasive nature of lumbar puncture procedures underscores the need for blood-based biomarkers, which remain underdeveloped. In contrast, peripheral blood is easily accessible and amenable to molecular analysis, making it a useful source for identifying biomarkers that may contribute to more precise assessment.

Recent advances in transcriptomic technologies have created new opportunities for blood-based biomarker development. RNA sequencing (RNA-seq), an open and unbiased platform, provides an in-depth view of the transcriptome from minimally invasive biofluids such as blood, and has been successfully applied to diagnosis, phenotyping, and prognosis in PD^15–19^. However, analyzing such high-dimensional transcriptomic data requires methods that can extract meaningful biological signal from tens of thousands of features relative to limited sample sizes. Conventional differentially expressed gene (DEG) analysis treats each gene independently and relies on arbitrary fold-change or p-value cutoffs, potentially overlooking coordinated regulatory networks that underlie disease processes^20^. To address these limitations, unsupervised approaches such as weighted gene co-expression network analysis (WGCNA) identify modules of co-regulated genes that capture systems-level transcriptional changes^21,22^, while contrastive principal component analysis (cPCA) isolates disease-specific variation by contrasting target samples against a reference background, with the distance from each sample’s projection to the background centroid providing a quantitative measure of molecular disease state^23^. Together, these network-based and contrastive methods enable the derivation of integrative molecular biomarkers that reflect coordinated pathophysiological processes rather than isolated gene-level alterations.

We hypothesized that a molecular trajectory score, derived from peripheral blood transcriptomics using unsupervised, data-driven methods, would independently predict long-term complications of PD. To test this, we employed WGCNA and cPCA to develop an objective biomarker for prognostic prediction in PD, based on RNA-seq data from the Parkinson’s Progression Markers Initiative (PPMI).

## Results

Baseline transcriptome data were obtained from 768 participants (PD = 579, HC = 189). Forty-seven subjects were excluded because their transcriptomic profiles were identified as outliers during RNA preprocessing. Consequently, 721 participants (PD = 541, HC = 180) were included in the final analysis. Demographic information is presented in Table 1. The PD and HC groups were comparable with respect to age at baseline and the proportion of male participants. Compared to the HC group, the PD group showed a higher burden of motor and non-motor features—except ESS—and lower putaminal DaT availability.

**Table 1:** Baseline characteristics of study participants

	HC	PD	*p* value
*N*	180	541	
Age at baseline	61.42 ± 11.37	61.64 ± 9.84	0.166
Sex, male	115 (63.9)	312 (57.7)	0.141
Education	16.11 ± 2.88	15.35 ± 3.52	0.004
Disease duration, years		1.26 ± 1.86	
Motor features			
MDS-UPDRS II	0.45 ± 0.97	6.54 ± 7.83	< 0.001
MDS-UPDRS III	1.27 ± 2.26	20.77 ± 9.65	< 0.001
Tremor subscore	0.03 ± 0.08	0.46 ± 0.34	< 0.001
PIGD subscore	0.02 ± 0.08	0.28 ± 0.32	< 0.001
Motor subtype			
Tremor dominant		341 (63.1)	
Indeterminate		59 (10.9)	
PIGD dominant		140 (25.9)	
HY stage	0.01 ± 0.11	1.65 ± 0.53	< 0.001
Nonmotor features			
MDS-UPDRS I	3.03 ± 3.01	6.50 ± 4.85	< 0.001
GdepS	1.34 ± 2.17	2.70 ± 2.79	< 0.001
STAI-Total	57.56 ± 14.44	67.83 ± 18.90	< 0.001
QUIP-CS	0.34 ± 0.81	0.53 ± 1.09	0.015
ESS	5.60 ± 3.40	6.21 ± 3.99	0.068
RBDSQ	2.87 ± 2.29	4.14 ± 2.81	< 0.001
SCOPA-AUT	5.99 ± 3.86	10.79 ± 6.99	< 0.001
MoCA	28.21 ± 1.10	26.77 ± 2.76	< 0.001
Baseline DaT			
Caudate	1.21 ± 0.35	0.81 ± 0.32	< 0.001
Putamen	1.53 ± 0.28	0.68 ± 0.26	< 0.001

Data are presented as mean ± standard deviation (SD) for continuous variables and n (%) for categorical variables. Continuous variables were compared using Student’s *t* test for normally distributed data or Mann–Whitney U test for non-normally distributed data. Categorical variables were compared using the chi-square test. Statistical significance was set at *P* < 0.05 (two-tailed).

*PD* Parkinson’s disease, *HC* healthy control, *HY* Hoehn and Yahr, *MDS-UPDRS* Movement Disorder Society-Unified Parkinson’s Disease Rating Scale, *MoCA* Montreal Cognitive Assessment, *RBDSQ* REM sleep behavior disorder screening questionnaire, *ESS* Epworth sleepiness scale, *GDepS* Geriatric depression scale, *STAI* state-trait anxiety inventory, *QUIP-CS* questionnaire for impulsive-compulsive disorders in Parkinson’s disease-current-short, *PIGD* postural instability and gait difficulty, *SCOPA-AUT* Scales for outcomes in Parkinson’s disease-Autonomic dysfunction; DaT, dopamine transporter.

### Construction of weighted correlation network

Of the 58,780 transcripts, 28,971 genes with a mean expression level greater than 4 were retained for downstream analysis. After outlier detection based on hierarchical clustering, 47 samples were identified as outliers and excluded from further analyses. Thus, a total of 28,971 genes from 721 participants were used to construct a weighted correlation network.

Subsequently, a scale-free topology criterion was employed to determine the soft-thresholding power, with *β* = 8 selected as the lowest value that resulted in an approximately scale-free network topology (Supplementary Fig. 2). The 28,971 genes were then clustered into 27 modules (Supplementary Fig. 3). Among 28,971 genes, 7533 genes (26.0%) were not assigned to any module (gray module) and were excluded from subsequent analyses.

### Identification of module–trait relationships and key modules

**Fig. 1:**
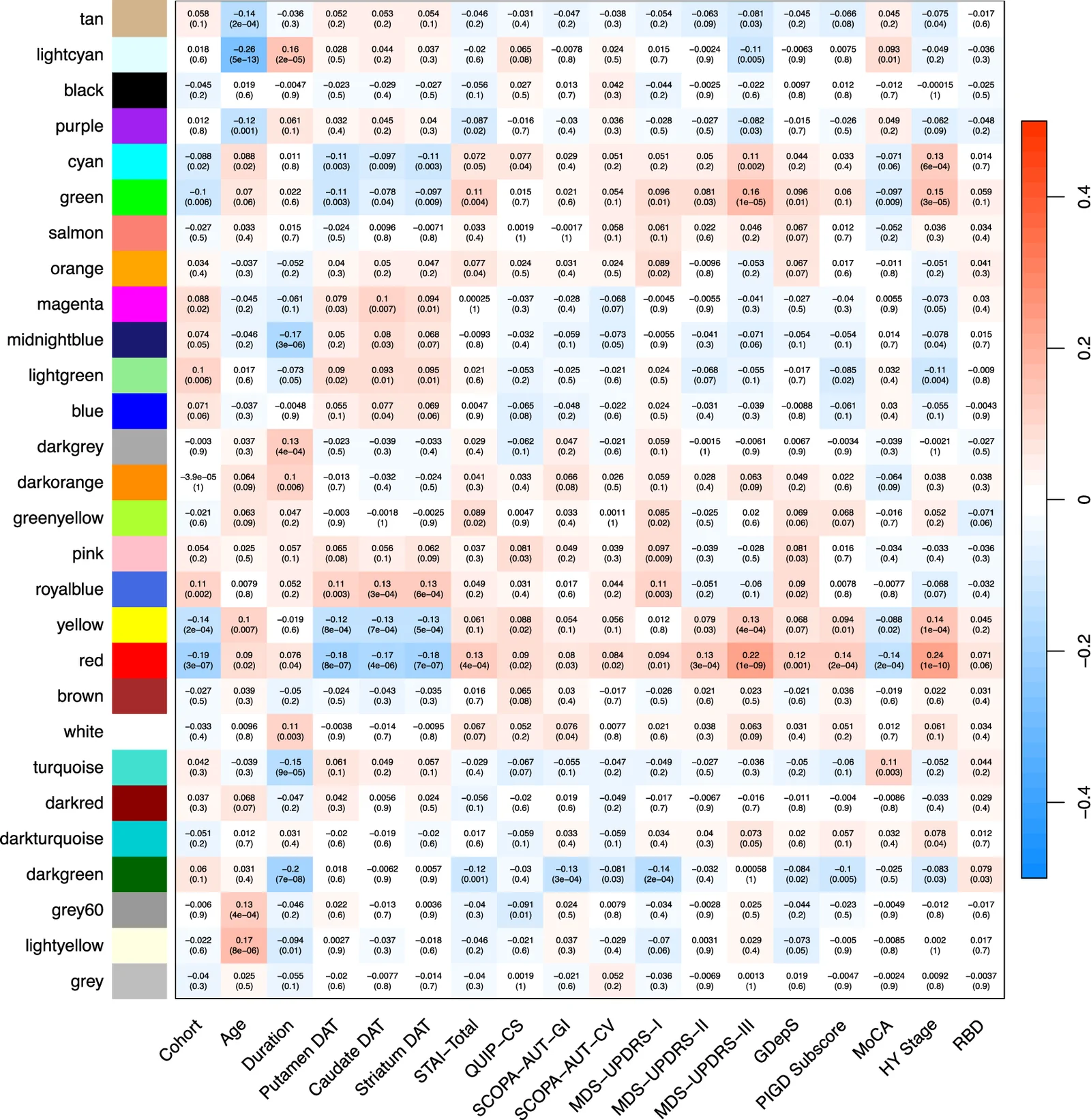
Heatmap presenting module – clinical features relationships. The yellow (*R* = –0.14, *P* = 2e–04) and red modules (*R* = –0.19, *P* = 3e–07) are significantly correlated with the disease status. These two modules show negative correlations with DaT binding ratios in the contralateral striatum (Putamen DAT, Caudate DAT, Striatum DAT), and MoCA scores. Furthermore, they are positively correlated with age, STAI, QUIP-CS, SCOPA-AUT, MDS-UPDRS-II/III, GDepS, PIGD subscore, and HY stage. DAT dopamine transporter, MoCA Montreal Cognitive Assessment, STAI State-trait anxiety inventory, QUIP-CS Questionnaire for Impulsive-Compulsive Disorders in Parkinson’s disease-Current-Short, SCOPA-AUT Scales for outcomes in Parkinson’s disease-Autonomic dysfunction, MDS-UPDRS Movement Disorder Society-Unified Parkinson’s Disease Rating Scale, GDepS Geriatric depression scale, PIGD Postural Instability and Gait Difficulty, HY Hoehn and Yahr, RBD REM sleep behavior disorder.

Among the 27 modules, the yellow (*R* = –0.14, *P* = 2e–04) and red (*R* = –0.19, *P* = 3e–07) modules demonstrated statistically significant correlations with study cohort status (PD = 1 vs. HC = 2). Additionally, both modules were significantly correlated with age, DaT imaging findings, QUIP-CS, SCOPA-AUT, MDS-UPDRS-II/III, GDepS, PIGD subscore, MoCA, and HY stage (Fig. 1). The yellow and red modules comprised 903 and 527 genes, respectively. A comprehensive list of genes included in each module is available in the Supplementary Data 1 and 2. Given that the purpose of WGCNA is to discover a gene expression signature related to PD and dimensionality reduction, these two modules were selected for further analysis.

**Fig. 2:**
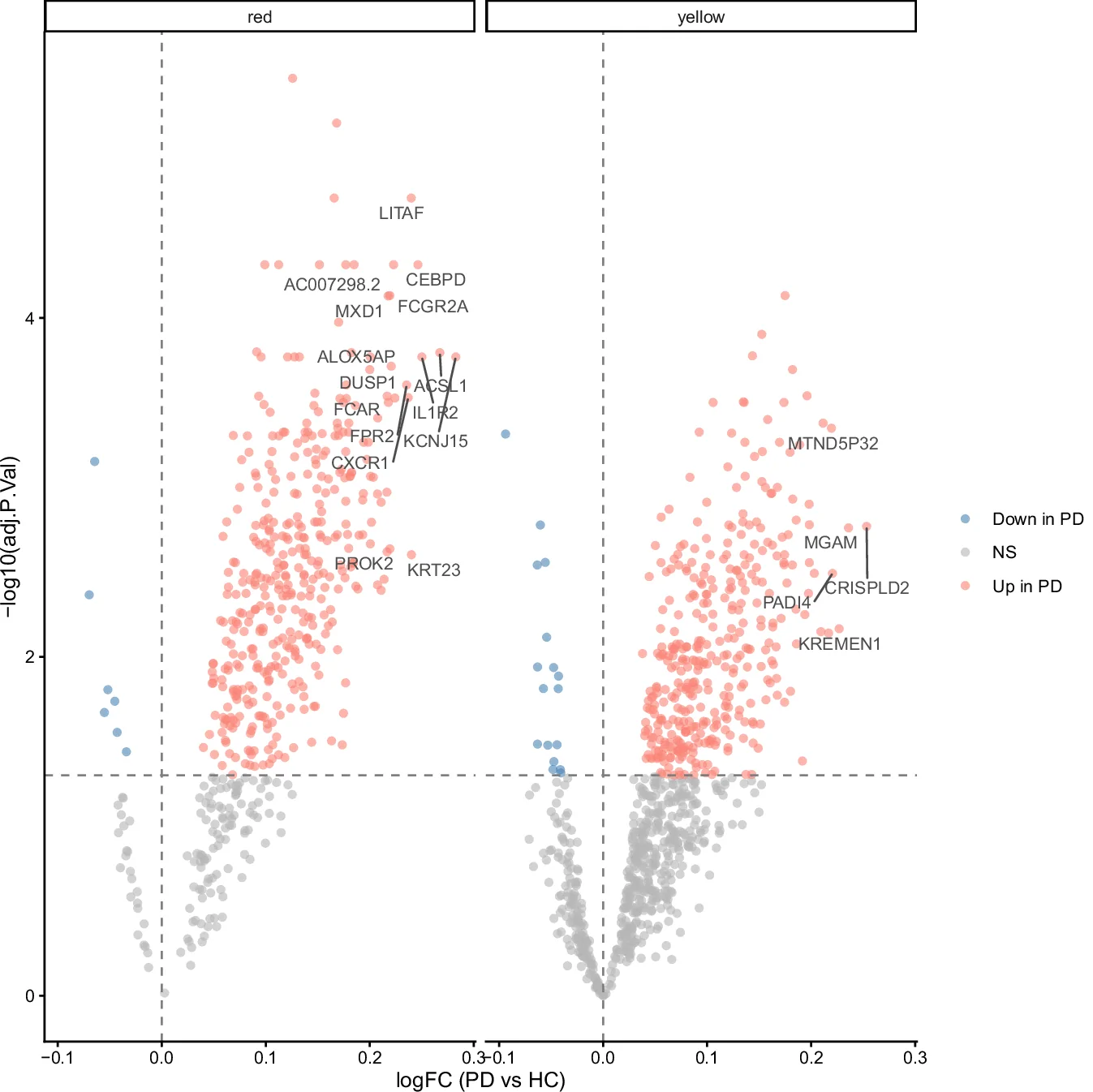
Differential expression analysis of yellow and red module genes between PD and HC participants. Volcano plots showing log2 fold-change (PD vs. HC, x-axis) and -log10 (adjusted *P* value, y-axis) for each gene within the red module (left) and the yellow module (right). Genes significantly upregulated in PD (FDR < 0.05, log2FC > 0) are shown in red; genes significantly downregulated in PD are shown in blue; non-significant genes are shown in gray. The top 20 most differentially expressed genes by absolute log fold change are labeled. The horizontal dashed line indicates the FDR threshold (0.05). FC fold change, adj.P.Val adjusted *P* value, NS not significant, PD Parkinson’s disease, HC healthy control, FDR false discovery rate.

DEG revealed that genes in both the red and yellow modules were predominantly upregulated in PD compared with HC, with very few significantly downregulated genes in either module (Fig. 2). The top differentially expressed genes in both modules were enriched for innate immune and neutrophil-related transcripts (Fig. 2, Supplementary Data 5).

### Module preservation analysis

Module preservation analysis was performed to validate the robustness of the identified co-expression modules. Among 25 modules identified in the training set (70% of samples), 22 modules demonstrated strong preservation (Zsummary >10) and 3 modules showed moderate preservation (Zsummary 2–10) in the independent test set (30% of samples). Notably, both the yellow module (Zsummary = 39.22) and red module (Zsummary = 29.26) exhibited strong preservation, indicating that the co-expression patterns underlying these disease-associated modules are highly reproducible and not artifacts of the specific sample composition (Supplementary Table 1).

### Enrichment analyses of genes in the yellow and red modules

**Fig. 3:**
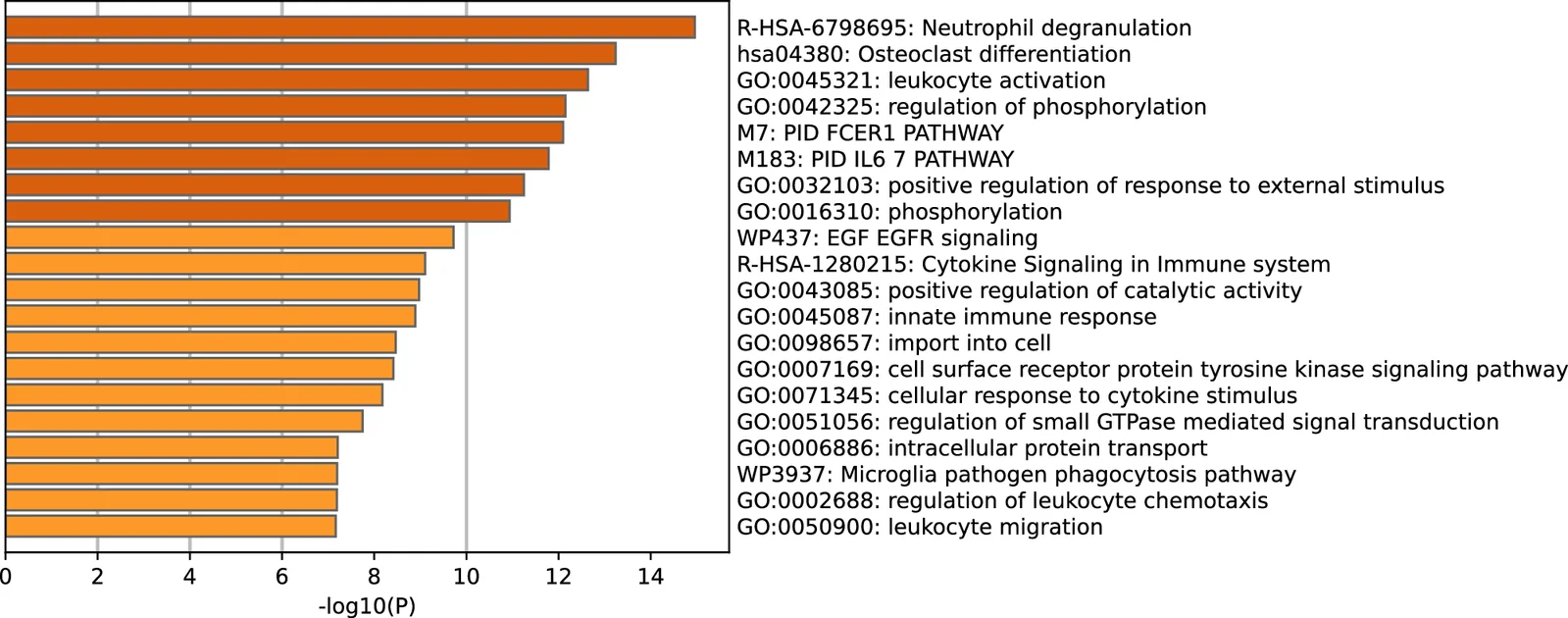
Enrichment analyses for genes of yellow and red modules. The top 20 clusters of enriched terms are visualized in descending order of *p* values. Most of the enriched ontology terms are related to inflammation, neutrophils, leukocytes, the immune system, and osteoclasts.

Enrichment analyses were conducted using over-representation analysis (ORA) for all 27 modules (Supplementary Data 6). The top 20 enriched terms of the yellow and red modules, which showed significant correlations with PD status and various clinical variables, were visualized in Fig. 3, ranked by descending p-values. A majority of the identified ontology terms were associated with inflammatory processes, neutrophils, leukocytes, the immune system, and osteoclasts. Detailed results, including gene lists and pathway/process terms, are provided in the Supplementary Data 1−4.

### Clinical and demographic correlates of PT scores

**Fig. 4:**
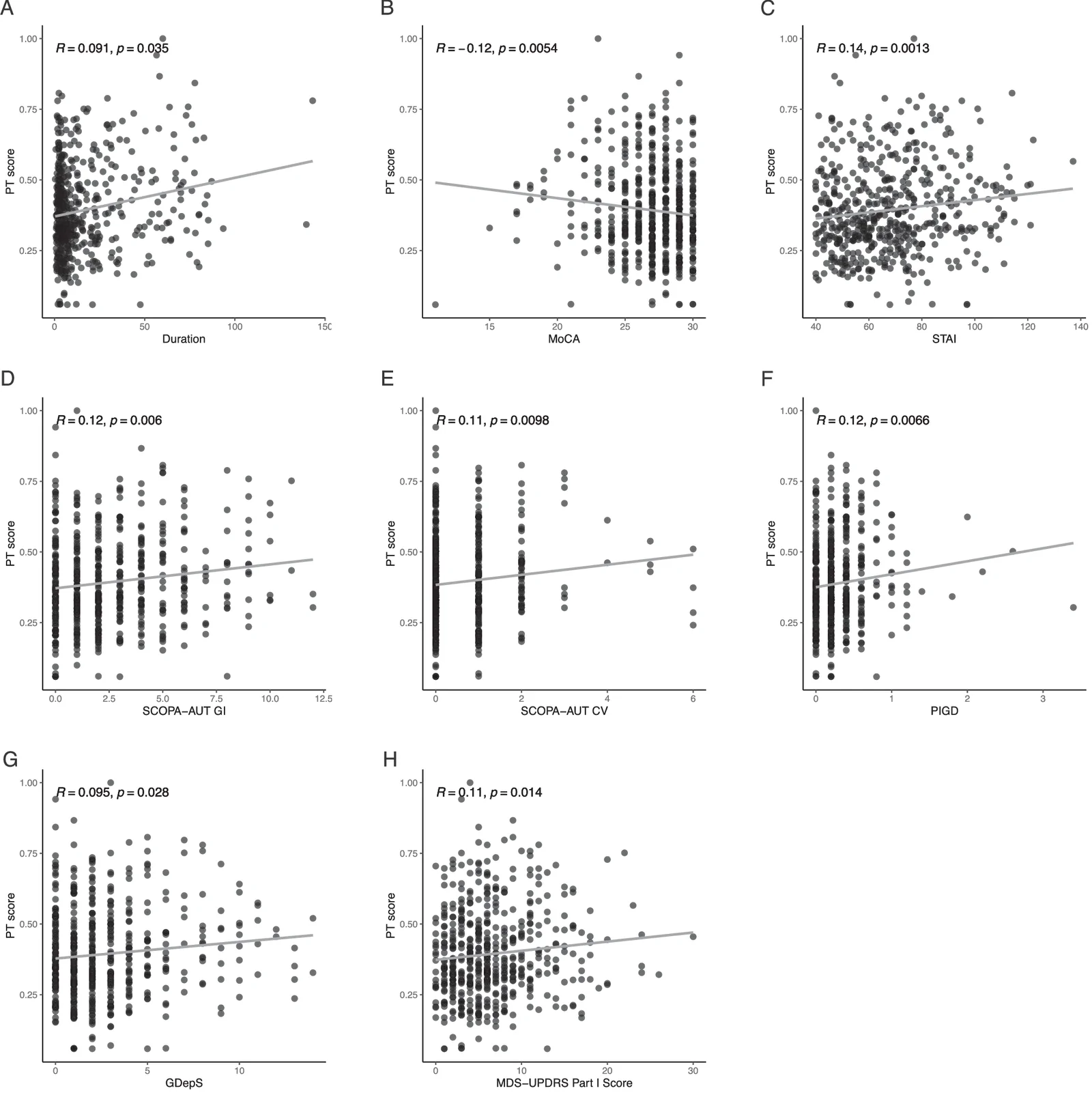
Correlations between the PT score and clinical features. PT scores are significantly correlated with disease duration (*R* = 0.091, *P* = 0.035, **A**), MoCA (*R* = –0.12, *P* = 0.005, **B**), STAI (*R* = 0.14, *P* = 0.001, **C**), SCOPA-AUT Gastrointestinal Score (*R* = 0.12, *P* = 0.006, **D**), SCOPA-AUT Cardiovascular Score (*R* = 0.11, *P* = 0.01, **E**), PIGD (*R* = 0.12, *P* = 0.007, **F**), GDepS (*R* = 0.095, *P* = 0.028, **G**), and MDS-UPDRS Part I Score (*R* = 0.11, *P* = 0.014, **H**). PT score, pseudo-temporal trajectory score. MoCA Montreal Cognitive Assessment, STAI State-trait anxiety inventory, SCOPA-AUT Scales for outcomes in Parkinson’s disease-Autonomic dysfunction, PIGD Postural Instability and Gait Difficulty, GDepS Geriatric depression scale, MDS-UPDRS Movement Disorder Society-Unified Parkinson’s Disease Rating Scale.

PT scores, representing the molecular disease trajectory relative to healthy controls, were computed using cPCA, which identifies components that capture variation enriched in the disease group compared to controls. PT scores were significantly correlated with disease duration (*R* = 0.091, *P* = 0.035, Fig. 4A), MoCA (*R* = –0.12, *P* = 0.005, Fig. 4B), STAI (*R* = 0.14, *P* = 0.001, Fig. 4C), SCOPA-AUT Gastrointestinal score (*R* = 0.12, *P* = 0.006, Fig. 4D), SCOPA-AUT Cardiovascular score (*R* = 0.11, *P* = 0.01, Fig. 4E), PIGD (*R* = 0.12, *P* = 0.007, Fig. 4F), GDepS (*R* = 0.095, *P* = 0.028, Fig. 4G), and MDS-UPDRS Part I Score (*R* = 0.11, *P* = 0.014, Fig. 4H). PT score showed only weak, non-significant positive correlations with RBDSQ scores (*P* = 0.077; Supplementary Fig. 4A), putaminal DaT availability (*P* = 0.76; Supplementary Fig. 4B), and MDS-UPDRS III (*P* = 0.28; Supplementary Fig. 4C).

### Prognostic value of PT scores for long-term complications

The median follow-up duration was 8.0 years (interquartile range: 4.9–12.0 years). During follow-up, 431 patients (79.7%) developed WO, 334 patients (61.7%) developed LID, 403 patients (74.5%) developed FOG, and 119 patients (22.0%) met criteria for cognitive impairment in PD patients. The median time to event was 3.6 years for WO, 6.1 years for LID, 4.7 years for FOG, and not reached for cognitive impairment.

Multivariable Cox regression analysis revealed that PT scores were significantly associated with the risk of cognitive impairment (HR = 7.31, 95% confidence interval [CI] = 2.06–25.91, *P* = 0.002) and FOG (HR = 3.43, 95% CI = 1.72–6.83, *P* < 0.001), independent of age, sex, disease duration, and putaminal DaT binding (Table 2). No significant association was observed between PT scores and WO (*P* = 0.679) or LID (*P* = 0.760). In contrast, decreased putaminal DaT binding showed a prognostic effect for WO (HR = 0.30, 95% CI = 0.19–0.48, *P* < 0.001) and LID (HR = 0.55, 95% CI = 0.34–0.89, *P* = 0.015).

**Table 2:** Cox proportional hazards regression analysis for long-term complications in Parkinson’s disease

Variable	Wearing off		Levodopa-induced dyskinesia		Freezing of gait		Cognitive impairment	
	HR	*P*	HR	*P*	HR	*P*	HR	*P*
Age	0.98 (0.97–0.99)	0.002	0.98 (0.97–1.00)	0.008	1.01 (1.00–1.02)	0.057	1.08 (1.06–1.11)	<0.001
Sex	1.07 (0.87–1.31)	0.531	0.86 (0.68–1.08)	0.185	1.32 (1.07–1.64)	0.010	1.09 (0.72–1.65)	0.684
Disease duration	1.28 (1.21–1.36)	<0.001	1.23 (1.15–1.32)	< 0.001	1.12 (1.05–1.19)	0.001	0.97 (0.85–1.11)	0.700
Putaminal DaT	0.30 (0.19–0.48)	<0.001	0.55 (0.34–0.89)	0.015	0.22 (0.13–0.36)	<0.001	0.22 (0.09–0.53)	<0.001
PT score	1.15 (0.60–2.18)	0.679	1.13 (0.52–2.46)	0.760	3.43 (1.72–6.83)	<0.001	7.31 (2.06–25.91)	0.002

Hazard ratios (HR) with 95% confidence intervals (CI) are presented. The PT score was modeled as a continuous variable (range: 0–1). All models were adjusted for age, sex, disease duration, and putaminal DaT binding ratio. The proportional hazards assumption was verified using Schoenfeld residuals. Statistical significance was set at *P* < 0.05 (two-tailed).

*PT score* pseudo-temporal trajectory score, *HR* hazard ratio, *CI* confidence interval, *WO* wearing-off, *LID* levodopa-induced dyskinesia, *FOG* freezing of gait, *DaT* dopamine transporter.

Internal validation using 1000-iteration bootstrap resampling confirmed the robustness of these prognostic associations of PT scores, with minimal bias in hazard ratio estimates (bias = 0.0331 for cognitive impairment; 0.0205 for FOG, Supplementary Table 2).

**Fig. 5:**
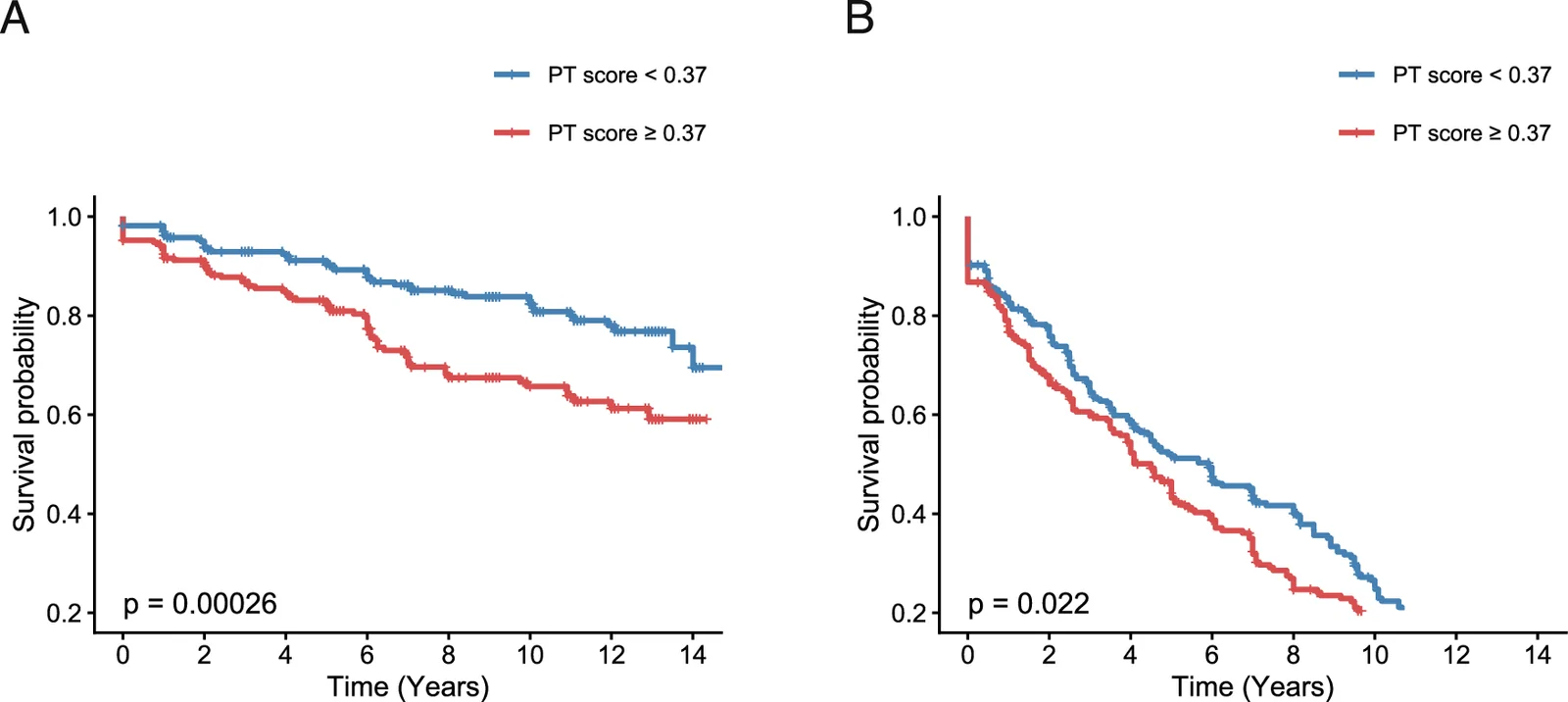
Kaplan-Meier survival curves for cognitive impairment and freezing of gait according to PT score. Patients were stratified by the median PT score (0.37). Patients with a higher PT score (≥0.37) demonstrated significantly lower event-free survival for both cognitive impairment (*p* = 0.00026, **A**) and FOG (*p* = 0.022, **B**) compared to those with a lower PT score (<0.37). Tick marks indicate censored observations. *P* values were calculated using the log-rank test. FOG freezing of gait, PT score, pseudo-temporal trajectory score.

Kaplan-Meier analysis demonstrated that patients with high PT scores (≥ 0.37, median) had significantly lower cognitive impairment-free survival (*P* < 0.001, Fig. 5A) and FOG-free survival (*P* = 0.022, Fig. 5B) compared to those with low PT scores (<0.37).

## Discussion

In this study, we employed a data-driven, unsupervised learning approach to derive a prognostic biomarker from peripheral blood transcriptome data in PD patients. Utilizing RNA-seq data from the PPMI cohort, we identified biologically relevant gene co-expression modules, interpreted their functional significance, and derived the PT score which reflects individual molecular disease trajectories. The prognostic utility of the PT score was subsequently assessed through survival analysis.

WGCNA enabled the construction of a scale-free gene co-expression network. Traditional unweighted networks define network adjacency based on gene expression similarity using hard thresholding, which may not accurately represent the continuous nature of co-expression. In contrast, the weighted network introduced in this study prevents information loss by assigning continuous values between 0 and 1 to the adjacency matrix through soft thresholding^24^. WGCNA revealed 27 gene modules. Among these, the yellow and red modules exhibited significant correlations with PD diagnosis and multiple clinical features, including DaT imaging findings and motor/non-motor symptom scores such as MDS-UPDRS-II/III, PIGD subscore, MoCA, GDepS, and HY scale. The dimensionality of the gene expression data was successfully reduced by selecting transcriptomes from these two relevant modules for further investigation.

Differential expression within the two PD-associated modules was characterized by predominant upregulation of innate immune and neutrophil-related transcripts in PD compared with HC, with minimal downregulation. This pattern aligns with the findings of Craig et al.^19^, who reported early and persistent upregulation of neutrophil-related gene expression in PD whole blood within the same PPMI cohort. Our network-based approach extends these gene-level findings by demonstrating that these immune transcripts form coordinated co-expression.

Functional enrichment analysis revealed that genes within the red and yellow modules were significantly involved in immune-related pathways, including neutrophil degranulation, leukocyte activation, M7: PID FCER1 PATHWAY, and M183: PID IL6 7 PATHWAY^25,26^. Along with the DEG results, these findings corroborate previous studies highlighting the role of both peripheral and central inflammation in the pathogenesis of PD^27^, especially regarding non-dopaminergic features^28–31^. In addition, the gene expression profiles of the two modules showed a significant association with osteoclast differentiation, which is of particular interest given the established link between abnormal bone metabolism and PD^32^. Accumulating evidence also suggests a role for noradrenergic neurotransmitter in bone metabolism, both directly^33^ and indirectly^34^.

To derive a quantitative molecular biomarker for PD, we applied cPCA to gene expression data from the red and yellow modules. This approach effectively distinguished PD-specific variation from that of healthy controls and quantified the proximity of each sample’s projection to the centroid of the healthy control projection, yielding a PT score that reflects disease trajectory at the molecular level. PT scores showed significant correlations with a range of motor and non-motor clinical assessments, including cognitive performance, mood symptoms, autonomic dysfunction, and the PIGD subscore in PD patients. While these correlations were modest in magnitude, they are consistent with the multifactorial nature of PD clinical phenotypes, where peripheral transcriptomic changes represent one of many contributors to clinical presentation. The pattern of associations—predominantly with non-motor and axial symptoms rather than classical dopaminergic features—suggests the PT score captures specific aspects of PD pathophysiology rather than overall disease severity.

A recent study has suggested that the noradrenergic subtype may represent a distinct biological phenotype of PD, characterized by gait disturbance, cognitive impairment, depression, and autonomic dysfunction^4^. Moreover, several studies have implicated the noradrenergic system in immune regulation and dopaminergic cell maintenance^35,36^. The pattern of clinical correlates observed with the PT score—predominantly non-motor symptoms and axial features—is consistent with the involvement of systems beyond the nigrostriatal dopaminergic pathway, potentially including noradrenergic pathways. However, we emphasize that this interpretation remains speculative without direct measurement of noradrenergic function, and the observed associations could also reflect other pathophysiological processes such as neuroinflammation or widespread neurodegeneration.

We further demonstrated that the PT score has prognostic value for specific long-term complications in PD, particularly cognitive impairment and freezing of gait. In contrast, PT scores were not predictive of other motor complications such as wearing-off or levodopa-induced dyskinesia, both of which are more tightly linked to nigrostriatal and intrastriatal mechanisms. Cognitive impairment and FOG in PD are thought to be associated with pathomechanisms beyond dopaminergic dysfunction and are commonly observed in the noradrenergic-dominant PD subtype^7^. Notably, higher PT scores were significantly associated with an increased risk of both cognitive impairment and FOG onset, even after adjusting for age, sex, disease duration, and putaminal DaT binding. These findings suggest that derived PT scores may reflect underlying pathophysiological processes complementary to nigrostriatal degeneration—such as neuroinflammation or noradrenergic dysfunction—which have been implicated in the development of cognitive and axial motor impairments in PD. This interpretation is further supported by the observation that PT scores were not associated with DaT availability or motor severity in this study (Supplementary Figure 4B, C). Importantly, although cross-sectional associations were significant (Fig. 4), their effect sizes were small, which may be attributed to the fact that the clinical manifestations of PD are shaped by diverse pathomechanisms, and that no single biomarker is likely to reflect any individual symptoms solely. Accordingly, the clinical utility of the PT score rests not on these modest cross-sectional correlations, but on the prognostic relationships for cognitive impairment and FOG which showed large and interpretable effect sizes.

Nevertheless, several limitations should be acknowledged. First, the analysis was based solely on the PPMI cohort, which may introduce cohort-specific biases and limits generalizability. Second, although we performed internal validation, the data-driven nature of the molecular biomarker still carries risk of overfitting and the influence of latent confounders. Future studies should aim to validate the PT score and its predictive capacity in independent cohorts and assess its integration with other biomarkers. Third, most study participants were Caucasian, further limiting the generalizability of the results. Validation in ethnically diverse populations is essential, as gene expression patterns may vary across ancestries. Fourth, our PD cohort included a small proportion of SAA-negative participants (*n* = 61, 11.3%), consistent with the rate reported in the broader PPMI^37^. Although we did not perform stratified analysis by SAA status due to the limited sample size in the SAA-negative subgroup, future studies with larger SAA-negative cohorts may further refine the interpretation of blood transcriptomic biomarkers in different biological subtypes of PD. Lastly, although we suggest noradrenergic system involvement based on clinical correlates, direct evidence linking the PT score to noradrenergic dysfunction is currently lacking. Future studies incorporating noradrenergic imaging or CSF norepinephrine measurements would be needed to test this hypothesis.

In conclusion, this study demonstrates the feasibility of deriving a molecular prognostic biomarker from peripheral blood transcriptomics in PD using data-driven, unsupervised methods. The PT score, derived from WGCNA-identified gene modules enriched for immune/inflammatory pathways and quantified via contrastive trajectory inference, showed potential prognostic value for cognitive impairment and freezing of gait, but not for levodopa-related motor complications such as wearing-off and dyskinesia. These findings suggest that peripheral transcriptomic signatures may capture aspects of PD pathophysiology extending beyond nigrostriatal dopaminergic degeneration, with relevance to long-term outcomes. However, external validation in independent cohorts is essential before clinical utility can be established, and the mechanistic basis of the PT score requires further investigation with direct measures of noradrenergic function.

## Methods

### Study design

**Fig. 6:**
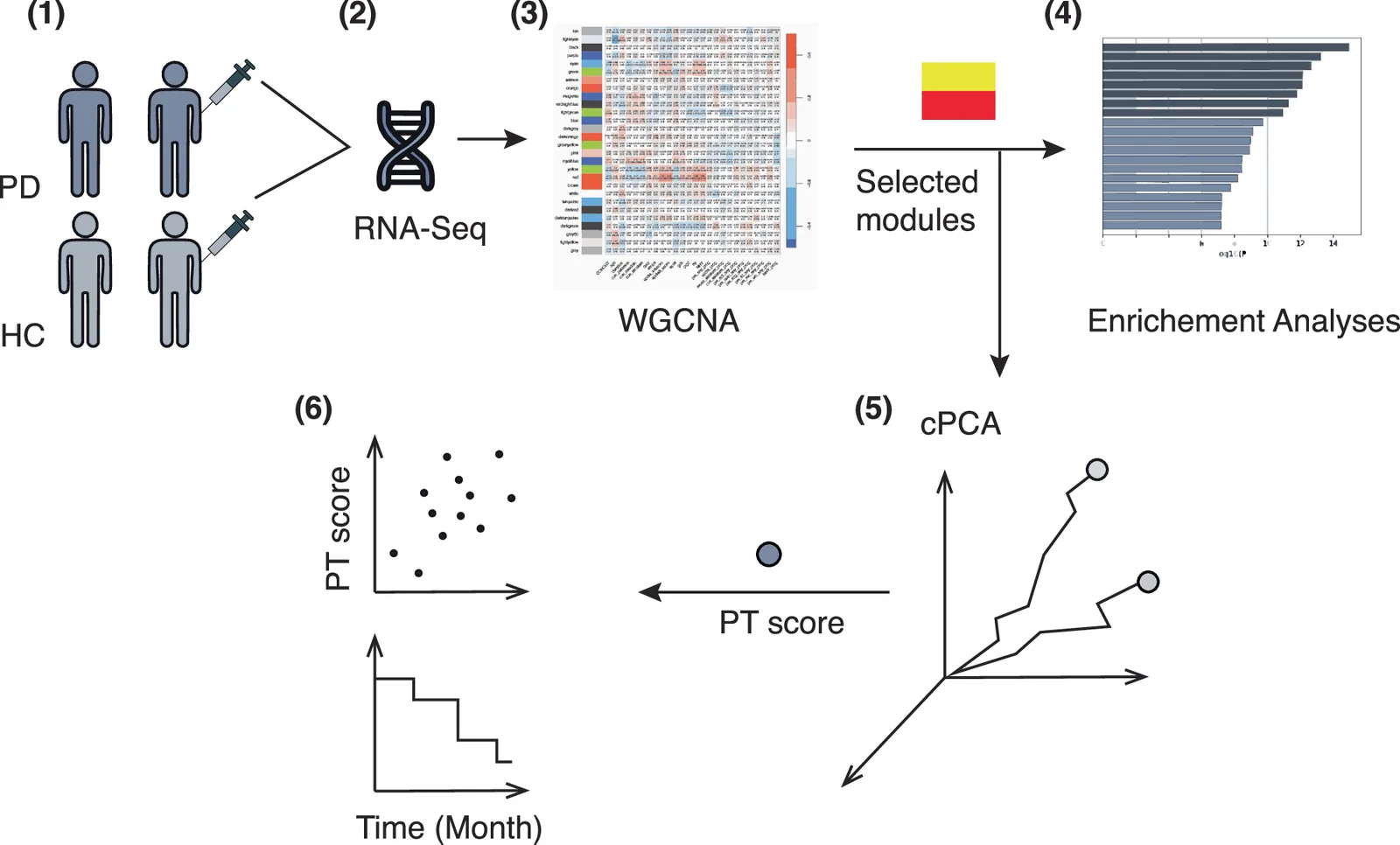
Study design. Blood samples were collected from participants with PD and HC (1). Preprocessing of RNA-seq data (2) were subjected to the WGCNA (3). Disease-associated modules were selected, and their gene expression data were used for enrichment analysis (4). cPCA was applied to module genes to derive PT score (5). Correlation and survival analyses were performed using the PT score (6). PD Parkinson’s disease, HC healthy control, WGCNA weighted gene co-expression network analysis, cPCA contrastive principal component analysis, PT score pseudo-temporal trajectory score.

The methodological workflow of this study comprised the following steps: (i) RNA-seq data preprocessing was conducted to ensure the data quality for subsequent analysis; (ii) the preprocessed data were subjected to WGCNA to identify disease-associated gene modules; (iii) enrichment analysis was then performed on the WGCNA-derived module genes to elucidate their biological significance; (iv) cPCA was applied to extract disease-associated patterns of module genes and derive a pseudo-temporal trajectory score for PD; (v) finally, survival analysis was performed to assess whether this trajectory score could predict long-term complications of PD. A schematic overview of our workflow is presented in Fig. 6.

### Participants and clinical variables

Baseline clinical and transcriptomic (RNA-seq) data of PD and healthy control (HC) participants were obtained from the PPMI (https://ida.loni.usc.edu/login.jsp?project=PPMI). We downloaded clinical data (March 19, 2026 release), and RNA-seq data (April 2, 2021 release), the most recent freeze available at the time of analysis. PPMI is a comprehensive, observational, international study designed to identify clinical, imaging, genetic, and biological markers of PD progression. It collects standardized data from early-stage PD patients, healthy controls, and individuals at risk of developing PD. The database includes longitudinal assessments of motor and non-motor symptoms, neuroimaging, biological samples, and genetic information, providing a robust framework for investigating disease mechanisms and potential biomarkers. Inclusion criteria for this study were as follows: (1) idiopathic PD and control participants whose RNA-seq data at baseline were available, (2) baseline assessment of clinical features and dopamine transporter imaging (DaT scan) were available. At baseline, age, sex, and disease duration since diagnosis of PD were assessed. For each patient, motor severity was assessed using HY (Hoehn and Yahr) stage, Movement Disorder Society-Unified Parkinson’s Disease Rating Scale (MDS-UPDRS) part II and III (MDS-UPDRS-II and MDS-UPDRS-III). Motor subscores and phenotypes were assessed according to the criteria derived from prior studies^38^. Overall cognition was assessed using Montreal Cognitive Assessment (MoCA) at screening visit. For non-motor symptoms, MDS-UPDRS part I (MDS-UPDRS-I), rapid eye movement sleep behavior disorder screening questionnaire (RBDSQ), Epworth sleepiness scale (ESS), Geriatric depression scale (GDepS), State-trait anxiety inventory (STAI), Questionnaire for Impulsive-Compulsive Disorders in Parkinson’s disease-Current-Short (QUIP-CS), Postural Instability and Gait Difficulty (PIGD, mean of MDS-UPDRS part II item 2.12, 2.13, part III item 3.10, 3.11, and 3.12), and Scales for outcomes in Parkinson’s disease-Autonomic dysfunction (SCOPA-AUT) were investigated at baseline. All data were collected following standardized protocols according to PPMI guidelines (https://www.ppmi-info.org), with appropriate ethical approvals and participant consent in accordance with the Declaration of Helsinki^39^. The current study is a secondary analysis of de-identified, publicly available PPMI data; therefore, no additional ethical approval was required.

### Definition of long-term complications

Long-term complications, including the development of wearing off (WO), levodopa-induced dyskinesia (LID), and FOG were assessed using MDS-UPDRS part IV, MDS-UPDRS-II, and MDS-UPDRS-III subitems (WO was defined as a score ≥1 on any of MDS-UPDRS part IV items 4.3, 4.4, 4.5, or 4.6; LID was defined as a score ≥1 on any of MDS-UPDRS part IV items 4.1 or 4.2; and FOG was defined as a score ≥1 on any of MDS-UPDRS-II item 2.13 or MDS-UPDRS-III item 3.11). Cognitive impairment in the PD dementia range was operationalized as a MoCA score below 21 at any follow-up visit^40^. Time-to-event was calculated from baseline assessment to the first occurrence of each complication, with participants censored at the last follow-up if the event did not occur.

### RNA-seq data preprocessing

A detailed description of the whole-transcriptome RNA-seq pipeline can be found at the PPMI repository. Since transcript count data generated via FeatureCounts is a widely used and general-purpose tool for read summarization, serving as input for various downstream analyses, we utilized the raw count data for our analysis.

To ensure suitability for network analysis, lowly expressed transcripts were filtered based on their mean expression values. Specifically, genes with variance-stabilized mean expression values greater than 4 were retained, corresponding to approximately the 50th percentile of expression across all transcripts. This threshold was selected to balance the retention of biologically relevant genes while excluding transcripts with expression levels too low for reliable correlation estimation, consistent with recommendations for WGCNA analysis^22^. The RNA-seq count data were then normalized and variance-stabilized using the vst() function implemented in the DESeq2 package (ver. 1.42.1). This transformation mitigates heteroscedasticity and yields a log2-like expression scale, rendering the data appropriate for correlation-based analyses such as WGCNA. Subsequently, sample outliers were identified using hierarchical clustering based on Euclidean distance with average linkage^22^. Visual inspection of the dendrogram (Supplementary Fig. 1) revealed a clear two-group separation, with an outlier branch separated from the main cluster at a height between 130 and 140. We selected 133 as the height of the cut (red line, Supplementary Fig. 1), which corresponded to 3 standard deviations above the mean pairwise distance. This excluded 47 outlier samples.

### Weighted gene correlation network analysis (WGCNA)

WGCNA was performed using the R package WGCNA, ver. 1.73^22^. Firstly, the co-expression similarity between gene i and j was defined as the correlation coefficient, ${S}_{{ij}}={cor}(i,{j})$. Using a soft-thresholding procedure, the co-expression similarity was transformed into a weighted adjacency matrix, ${a}_{{ij}}={{S}_{{ij}}}^{\beta }({where}\beta > =1)$, allowing weights to range continuously between 0 and 1 rather than being restricted to binary values (0 or 1). By raising the correlation to a power, the disparity between strong and weak correlations can be amplified. To select an appropriate power term, the scale-free topology fitting method was tested across a range of power values from 1 to 20. Because there is a trade-off between mean connectivity (maintaining mean number of connections) and maximizing scale-free topology fit, the lowest power value at which the saturation of scale-free topology above 0.80 was chosen, as recommended^41^. The resulting adjacency matrix was then transformed into a topological overlap matrix (TOM), which incorporates both topological structure and gene expression similarity^42,43^, and subsequently fed into unsupervised hierarchical clustering to identify gene modules—clusters of highly interconnected genes.

Following module identification, the module eigengene (ME)—a representative value characterizing each module—was defined as the first principal component of gene expression within the module. This provides a sample-level summary that captures the predominant expression trend of each module. To identify relevant modules, relationships between module gene expression and clinical features were estimated using correlations between MEs and various clinical variables. Modules showing significant correlations may represent biologically meaningful pathways related to the clinical characteristics^22^.

### Internal validation of module stability

To assess the reproducibility of the identified co-expression modules, we performed module preservation analysis using a split-sample approach. The dataset was randomly divided into training (70%) and test (30%) sets. WGCNA was performed on the training set to identify gene modules, and module preservation statistics were calculated in the test set using the modulePreservation() function in the WGCNA R package with 200 permutations^44^. The composite preservation statistic Zsummary, which integrates density-based and connectivity-based preservation measures, was used to evaluate module robustness. Following established guidelines, Zsummary > 10 indicates strong evidence of preservation, Zsummary 2–10 indicates moderate preservation, and Zsummary < 2 suggests no preservation^44^.

### Differential expression analysis

To characterize the biological directionality of WGCNA co-expression modules, differential gene expression analysis was performed comparing PD patients and HC. VST (variance-stabilizing transformation)-normalized expression data, used for co-expression network construction, were analyzed using the limma package (ver. 3.58.1) in R. A design matrix without intercept was constructed specifying group membership (PD and HC), and a contrast of PD minus HC was defined to identify genes with differential expression. Empirical Bayes moderation was applied using the eBayes function to stabilize variance estimates across genes. Differentially expressed genes (DEGs) were identified using a false discovery rate (FDR)-adjusted *p* value threshold of <0.05 (Benjamini-Hochberg correction). For the red and yellow modules, the proportion of significantly up- and downregulated genes (positive and negative log fold change, respectively) was summarized to determine the overall direction of module regulation in PD relative to HC. Results were visualized using volcano plots, with the top 20 DEGs ranked by absolute log fold change labeled.

### Enrichment analyses for key modules

To elucidate the biological significance of the identified gene modules, pathway/process enrichment analysis was performed using Metascape (http://metascape.org). Gene lists for each module were submitted to the platform, and the analysis incorporated several ontology categories, including Gene Ontology (GO) Biological Process, Molecular Function, and Cellular Component, as well as Hallmark Gene Sets, Reactome Gene Sets, KEGG Pathways, Canonical Pathways, and BioCarta Gene Sets. A standard accumulative hypergeometric statistical test was employed to identify significantly enriched ontology terms. To reduce redundancy, resultant terms with a p-value < 0.01, a minimum hit count of 3, and an enrichment factor > 1.5 were automatically clustered based on semantic similarity (Kappa similarity > 0.3). The most enriched pathway within each cluster was designated as the representative term^45^.

### Pseudo-temporal trajectory score estimation and clinical relevance in PD

Individual disease trajectory scores were estimated in patients with PD using Contrasted Trajectory Inference (cTI) implemented in the NeuroPM-box^46^. This context-aware, unsupervised approach applies cPCA to identify disease-associated patterns that are enriched in the PD cohort compared to controls, and computes a pseudo-temporal trajectory score (PT score) that reflects each subject’s relative disease progression stage^23^.

Briefly, expression matrix of identified disease-associated modules genes was embedded into a reduced contrastive space. PD samples served as the target dataset and HC samples as the background dataset, allowing extraction of variance components enriched in PD relative to HC. In the resulting contrastive principal component (PC) space, the centroid of HC samples was computed as the reference point representing the unaffected molecular state. The Euclidean distance from each PD sample’s position to this HC centroid was then calculated, providing a continuous measure of each sample’s molecular deviation from the healthy reference. Distances were min-max scaled to the range [0, 1], yielding the final PT score. Higher PT scores indicate greater molecular deviation from the HC reference (i.e., a more advanced molecular state), while lower scores indicate closer proximity to the HC molecular profile.

### Statistical analysis

Continuous variables are presented as mean ± standard deviation (SD). Comparisons of continuous variables were performed using Student’s *t* test or Mann–Whitney U test, as appropriate. Categorical variables were compared using the chi-square test. Pearson’s or Spearman’s correlation coefficients were calculated to evaluate associations between PT scores and clinical variables. Univariable and multivariable Cox proportional hazards regression analyses were performed to assess the prognostic significance of the PT score regarding long-term complications including WO, LID, FOG, and cognitive impairment with age, sex, disease duration, and putaminal DaT availability as covariates in PD. Kaplan-Meier survival curves were constructed to estimate event-free survival probabilities, and the log-rank test was used to compare survival distributions between groups stratified by the median PT score. All statistical analyses were conducted using R software (version 4.3.3), and a two-tailed P-value < 0.05 was considered statistically significant.

## Supplementary information

Supplementary Information_revised_260704.

Supplementary Data_revised_260704.

## Data Availability

The data that support the findings of this study are available from the Parkinson’s Progression Markers Initiative (PPMI) database but restrictions apply to the availability of these data, which were used under license for the current study, and so are not publicly available. Access to PPMI data can be requested at https://www.ppmi-info.org/access-data-specimens/download-data.

## Code availability

The R code for WGCNA and statistical analyses is available from the corresponding author upon reasonable request.
