# Supplementary material for: Peripheral blood transcriptomic signature is associated with cognitive impairment and freezing of gait in Parkinson’s disease: a data-driven approach
Source: NPJ Parkinsons Dis. 2026 Aug 15;12:212. doi: 10.1038/s41531-026-01527-0 (PMC13542282; doi:10.1038/s41531-026-01527-0)
Supplement: Supplementary file 1 [file 41531_2026_1527_MOESM1_ESM.pdf]

Supplementary Figure 1. Sample clustering dendrogram for outlier detection in WGCNA.

Hierarchical clustering based on Euclidean distance with average linkage was performed on the normalized RNA-seq expression matrix. The red horizontal line indicates the cutoff at 3 standard deviations (SD) above the mean clustering height (cutHeight = 133), used as the primary outlier exclusion threshold; the blue line indicates the 2 SD threshold (cutHeight = 114) with one additional sample.

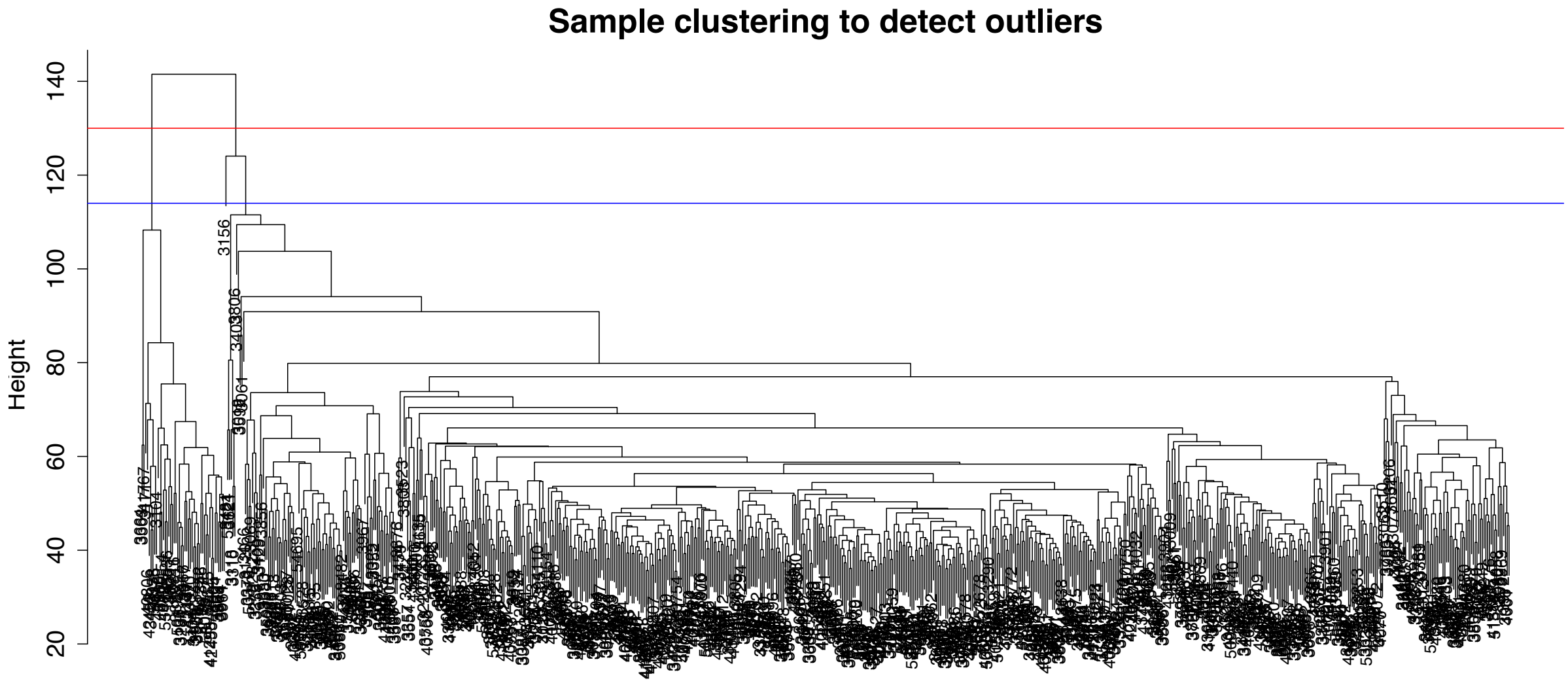

Supplementary Figure 2. Scale free topology model fitting and mean connectivity by raising the power value from 1 to 20.

Since there is a trade-off between scale-free topology model fit and a high mean number of connections, the lowest power at which the saturation of scale-free topology above 0.80 is 8 was picked to prevent loss of information.

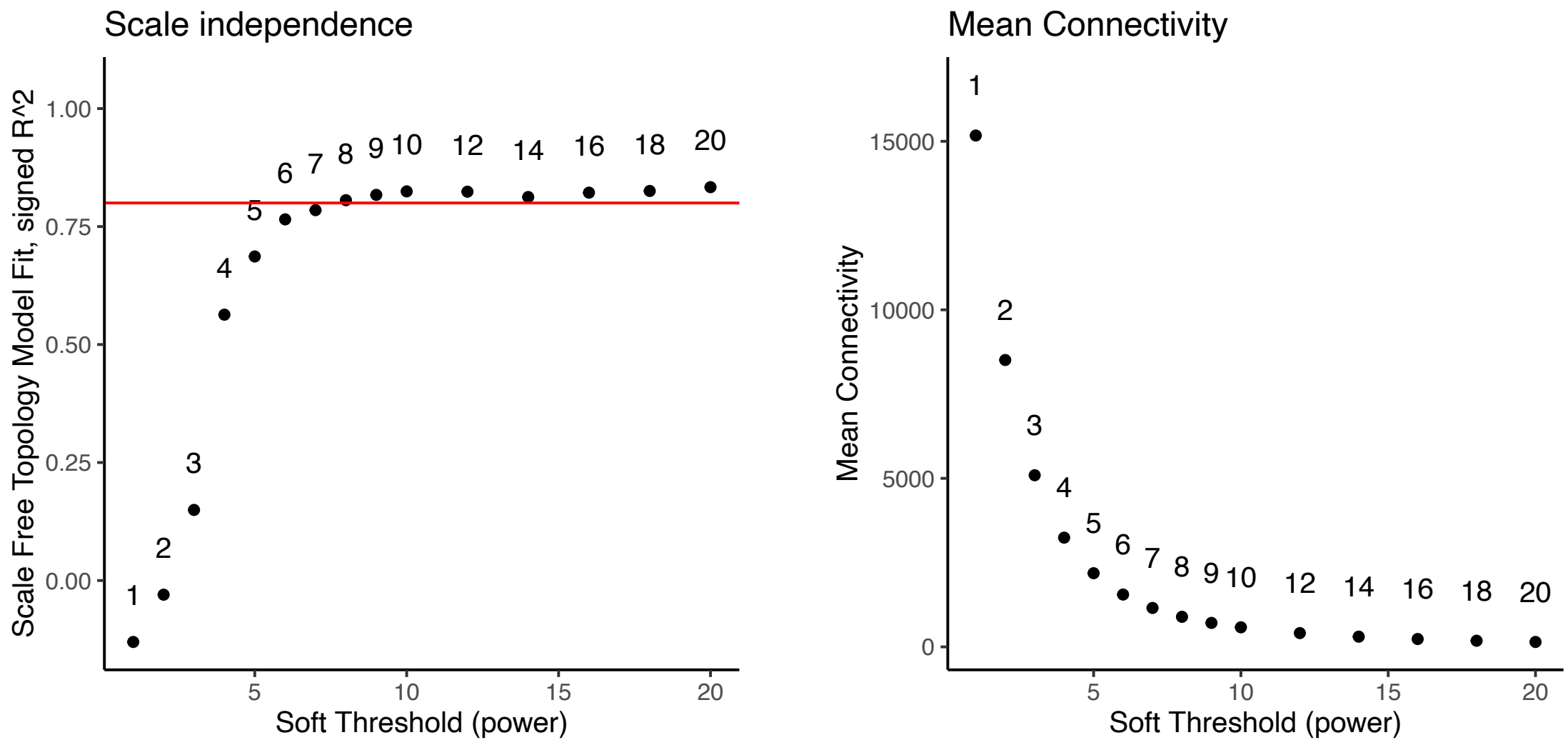

Supplementary Figure 3. Hierarchical cluster dendrogram to divide genes into modules.

28,971 genes are divided into 27 modules (number of genes); black (498), blue (4732), brown (3847), cyan (124), darkgreen (57), darkgrey (45), darkorange (33), darkred (85) darkturquoise (51), green (543), greenyellow (181), grey60 (105), lightcyan (117), lightgreen (94), lightyellow (88), magenta (326), midnightblue (119), orange (39), pink (427), purple (236), red (527), royalblue (87), salmon (126), tan (173), turquoise (7845), white (30), and yellow (903) modules by hierarchical clustering algorithm. 7,533 genes that are not assigned to any module are grouped into the grey module.

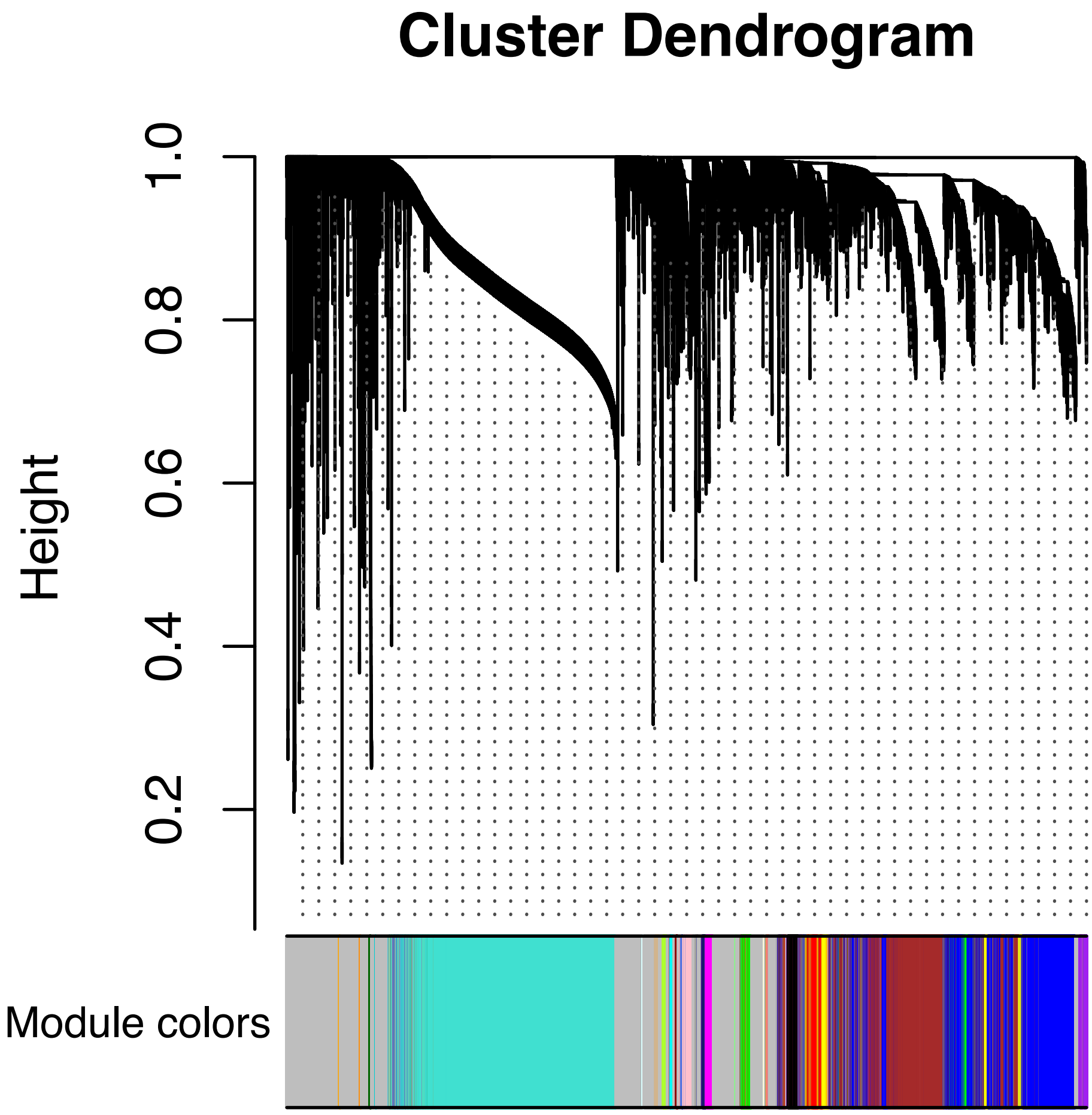

Supplementary Figure 4. Clinical correlates of the PT score.

PT scores show no significant correlations with RBDSQ score (A), Putaminal SBR (B), and MDS-UPDRS III (C). Each dot represents a single PD participant.

PT score, pseudo-temporal trajectory score; RBDSQ, REM sleep behavior disorder screening questionnaire; SBR, striatal binding ratio; MDS-UPDRS, Movement Disorder Society-Unified Parkinson's Disease Rating Scale

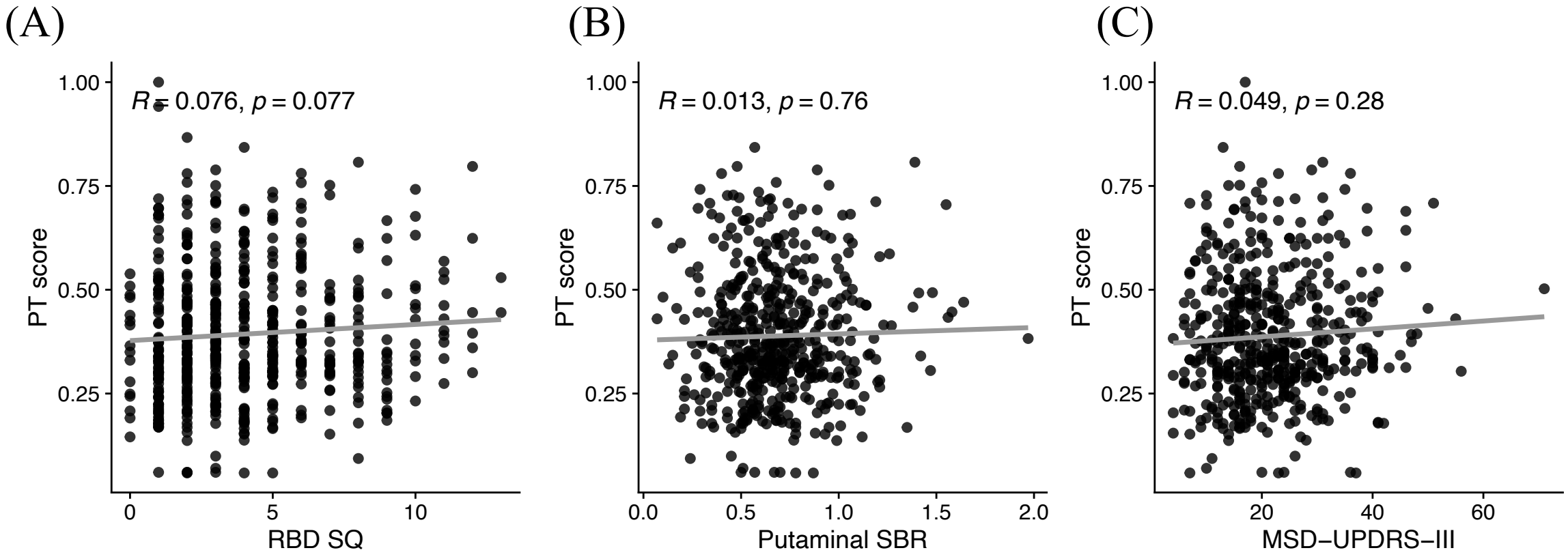

Supplementary Table 1. Module Preservation Analysis Results

Module	Zsummary	Preservation
turquoise	64.36	Strong
brown	42.78	Strong
blue	39.66	Strong
**yellow**	**39.22**	**Strong**
green	34.38	Strong
**red**	**29.26**	**Strong**
black	27.38	Strong
royalblue	26.78	Strong
tan	25.14	Strong
pink	22.24	Strong
magenta	20.99	Strong
cyan	20.94	Strong
salmon	18.30	Strong
purple	18.08	Strong
greenyellow	16.49	Strong
lightcyan	15.05	Strong
darkgreen	14.67	Strong
midnightblue	12.82	Strong
grey60	12.21	Strong
lightyellow	12.17	Strong
lightgreen	11.63	Strong
darkturquoise	11.13	Strong
darkred	9.48	Moderate
darkgrey	9.34	Moderate
orange	9.07	Moderate

Module preservation was assessed using a 70:30 train-test split. WGCNA was performed on the training set, and preservation statistics (Zsummary) were calculated in the test set. Zsummary > 10 indicates strong preservation; Zsummary 2–10 indicates moderate preservation. White and darkorange modules are absent from this table because they were too small to be detected in the training set and only emerged when the full dataset (721 samples) was used. Yellow and red modules (bold) were selected for downstream analysis based on significant correlations with PD status and clinical features.

PD, Parkinson's disease; WGCNA, weighted gene correlation network analysis.

Supplementary Table 2. Bootstrap validation of the PT score for long-term clinical outcomes

Outcome	N	Original HR (95% CI)	Bootstrap HR (95% CI)	Bias
Cognitive impairment	119 (22.0%)	7.31 (2.06–25.91)	7.53 (2.54–31.03)	0.0331
Freezing of gait	403 (74.5%)	3.43 (1.72–6.83)	3.55 (1.77–7.44)	0.0205

HR, hazard ratio; CI, confidence interval. Bootstrap-corrected HRs and bias estimates were derived from 1,000 resampling iterations of the full cohort. Bias represents the difference between the mean bootstrap HR estimate and the original HR on the log scale. All models were adjusted for age, sex, disease duration, and putaminal DaT (dopamine transporter) binding.
